# Thymic B Cells as a New Player in the Type 1 Diabetes Response

**DOI:** 10.3389/fimmu.2021.772017

**Published:** 2021-10-21

**Authors:** Richard B. Greaves, Dawei Chen, E. Allison Green

**Affiliations:** Centre for Experimental Medicine and Biomedicine, Hull York Medical School, University of York, York, United Kingdom

**Keywords:** type 1 diabetes, thymic B cells, autoimmunity, computational modelling, negative selection

## Abstract

Type 1 diabetes (T1d) results from a sustained autoreactive T and B cell response towards insulin-producing β cells in the islets of Langerhans. The autoreactive nature of the condition has led to many investigations addressing the genetic or cellular changes in primary lymphoid tissues that impairs central tolerance- a key process in the deletion of autoreactive T and B cells during their development. For T cells, these studies have largely focused on medullary thymic epithelial cells (mTECs) critical for the effective negative selection of autoreactive T cells in the thymus. Recently, a new cellular player that impacts positively or negatively on the deletion of autoreactive T cells during their development has come to light, thymic B cells. Normally a small population within the thymus of mouse and man, thymic B cells expand in T1d as well as other autoimmune conditions, reside in thymic ectopic germinal centres and secrete autoantibodies that bind selective mTECs precipitating mTEC death. In this review we will discuss the ontogeny, characteristics and functionality of thymic B cells in healthy and autoimmune settings. Furthermore, we explore how *in silico* approaches may help decipher the complex cellular interplay of thymic B cells with other cells within the thymic microenvironment leading to new avenues for therapeutic intervention.

## Introduction

Type 1 diabetes (T1d) is an autoimmune condition characterised by the destruction of the insulin producing beta (β) cells in the islets of Langerhans by co-operative interaction between the innate and adaptive immune systems; the final assault being perpetuated by CD8^+^ cytotoxic T cells (1–3). The non-obese diabetic (NOD) mouse that spontaneously develops T1d in a manner thought to be similar to man, has been an important resource in analysing the complexity of the T1d immunopathology (4). In line with the important role that the thymus plays in purging thymocytes with autoreactive T cell receptors (TcRs) (5), T1d progression is linked to defects in central tolerance increasing the output of islet-reactive T cells (6, 7), although definitive understanding of why negative selection of islet-reactive T cells occurs remains elusive.

Studies in NOD mice deficient in B cells have revealed an initially unappreciated role for B cells not only in helping the CD4^+^ T cell response to islet antigens (8–10), but also promoting the survival of islet-reactive CD8^+^ CTL *in situ* (11). This important role for B cells in the T1d process has been translated to man, where T1d progression risk is determined by the number of serum antibodies to islet antigens (12) and evidence that T1d severity is characterised by increased infiltration of islets letion of B cells in people newly diagnosed with T1d results in transient remission of the condition (14).

Recently, we reported a new role for B cells in the T1d process- mediators of breakdown in thymic central tolerance (15). We showed that thymic B cells, normally a minor constituent of a healthy thymus in both man and mouse (16, 17), rapidly increase following the initiation of islet infiltration with immune cells (termed insulitis) but prior to overt clinical manifestation of T1d. Further, we showed that thymic B cells reside in putative thymic germinal centres, undergo *in situ* class switching and differentiation into plasma cells. Autoantibodies from these plasma cells targeted specific medullary thymic epithelial cells (mTECs) for antibody-mediated apoptosis, leading to increased output of T cells that bypassed negative selection thereby enhancing T1d progression. Our finding was reminiscent of the immunopathology of the autoimmune conditions of myasthenia gravis (18) and systemic lupus erythematosus (19, 20), suggesting that thymic B cell abnormalities may be a common link between certain autoimmune conditions.

Analysis of thymic B cells is challenging; thymic involution as we age necessitates human thymic studies to be largely restricted to foetal or paediatric tissue which, quite rightly, is ethically sensitive to procure. Although it could be argued the availability of murine thymi negates issues of thymic cellularity during the involution process, researchers have an ethical responsibility to minimise the size of murine cohorts undergoing experimental procedures. Systems biology, which incorporates the development of computational models that can recapitulate the complexity of the immune response in defined tissues- offers an attractive approach to evaluate the molecules and signal pathways that contribute to the thymic B cell-mediated progression to T1d.

Examples of existing simulation studies in biology are numerous: granuloma formation (21–23), breast cancer metastasis (24) and lymphoid tissue formation (25, 26). There has also been a considerable body of work on modelling epithelial tissues (27, 28). Following this precedent it will be instructive to create a computer simulation of the early thymic events in T1d development.

This review is, therefore, structured in two parts; first we will discuss our current understanding of the ontogeny, phenotype and function of thymic B cells in health and disease, and secondly, we review the development and challenges of the systems biology approach of generating a computational model of thymic autoimmunity.

## Ontogeny of Thymic B Cells

Thymic B cells were initially discovered during immunohistochemical studies of human thymic tissue (17) and subsequently in mice (29, 30).Thymic B cells, a minor population of cells within the thymic cellular pool, are present from embryonic age through to adulthood, the number of cells remaining stable throughout life, although reports of the ageing murine thymus suggest increasing numbers of thymic B cells are a characteristic of the thymic involution (6, 7, 31). Since the discovery of thymic B cells, the origin of the cells- *in situ* development versus recirculation from the periphery- has been debated. For example, Sato et al., demonstrated that peripheral B cells preferentially migrate to the thymus in response to increasing levels of intrathymic B lymphocyte chemoattractant CXCL13, although it was noted this occurred in aged mice (32). In contrast, parabiosis models argue against peripheral B cell migration to the thymus (33). Our adoptive transfer studies in NOD mice, revealed splenic B cells had minimum capacity to migrate to the thymus even when transferred at the post-insulitic, pre-diabetic phase when thymic B cell numbers rapidly increase (Davis and Green, unpublished observations). Interestingly, B cells isolated from the thymus almost exclusively migrated back to this organ following adoptive transfer into recipient mice. These findings related to peripheral B cell thymic migration, align with others, and suggest that thymic B cells originate from *in situ* development (34, 35). Further support for this hypothesis was provided by studies in NOD-RAG2p-GFP reporter mice (15), that enable monitoring of recombination activating gene (RAG) activity in developing B and T cells (36). Comparison of thymic B cell RAG activity in NOD mice revealed a significant increase in rearrangement of the B cell receptor (BcR) in the post-insulitic, pre-diabetic phase compared to pre-insulitic phase (15). Interestingly, the level of thymic B cell RAG activity was similar between NOD mice in the pre-insulitic phase to the levels seen for control, age-matched mice, and in this latter strain thymic B cell RAG activity remained at a constant level as mice aged. Although it could be speculated that increased RAG activity in thymic B cells may represent aberrant re-ignition of RAG genes in peripheral B cells that migrate to the thymus and potentially undergo receptor editing of the BcR, studies by Gay et al., demonstrating that peripheral RAG^-^ B cells were unable to reactivate RAG following a series of mitogenic and antigenic B cell stimulations, argue against this possibility (37). Similar findings looking at the ability of immunisation to re-ignite RAG activity supports the hypothesis that RAG expression in B cells is restricted to their developmental stages (38, 39). Thus, the thymus of mice *per se* can support B cell development, but in the context of T1d, there is an acceleration in B cell development specifically during the post-insulitis, prediabetic phase, and this coincides with a rapid increase in thymic B cell numbers (15).

If thymic B cells develop *in situ*, what are the progenitor cells and signal pathways involved? Although it is well established that the thymus is seeded with common lymphoid progenitor cells (CLP) that have T, B and NK cell potential (40), it has been challenging to map the thymic B cell development pathway assuming it originates from the CLP population. Several studies have identified B lineage-committing transcription factors and cells within the thymus that have a phenotype akin to B cell committed precursors in their natural developmental habitat, the bone marrow (34). In addition, McKenna et al., demonstrated that addition of bone marrow isolated pro-B cells into thymic organ cultures stimulated with Fms-related receptor tyrosine kinase 3 (FLT3) and IL-7 induced immature B cell development (41). Interestingly, similar culture of pro-B cells with cell-lines derived from bone marrow or thymic stroma cultured did not induce complete B cell development in the presence of FLT3 and IL-7. However, these studies are based on B cell committed progenitors, that is pro-B cells, and it has been far more challenging to identify within the thymus the cell(s) that lie upstream of the pro-B cell that have been identified in the bone marrow (42, 43).

As well as speculation on the definitive progenitor from which intrathymic B cells develop, there also been controversy in the signal pathways that lead to intrathymic B cell development. For example, it has been proposed that intrathymic B cell development may be a default pathway resulting from perturbation of the T cell developmental pathway due to impaired CD3 or T cell receptor β (TcRβ) signalling (44) or inappropriate Notch signalling (45). However, Feyerabend et al., using the cre-lox system to delete Notch in the DN1 population, a population that has both B and T cell potential, did not divert development down the B cell developmental pathway (46). More understanding is required as to both the progenitor and signal pathways that enable the development of thymic B cells. Such studies will be invaluable in determining whether increased thymic B cell development characteristic of T1d progression in NOD mice relates to perturbation of these signal pathways.

## Phenotype of Thymic B Cells

B cells can be divided into several classifications: follicular (FO), marginal zone (MZ), peritoneal (B1a) and regulatory (Bregs). Each subgroup of B cells can be identified by their surface markers, and each has particular functions to play in the immune response. Although Bregs have been identified in the thymus (47) Bregs will be discussed elsewhere in this Special Edition and will not be considered here. MZ B cells have received particular attention in the autoimmune setting, due to an aberrant increase in both their numbers and location in murine autoimmune models (48–50) as well as in humans living with certain autoimmune conditions (51, 52). Innate-like MZ B cells express high levels of IgM and low levels of IgD alongside coexpression of CD21 and CD35 (53). They can function to remove apoptotic cell debris impeding autoimmunity (54), or conversely, they may promote autoimmunity *via* their polyreactive receptors (48). Furthermore, weak signalling through the BcR (55, 56) or perturbation of the negative regulators of BcR signalling- including FcγRIIB- promotes expansion of MZ B cells that are more efficient at presenting antigen to T cells (57), including potentially autoreactive T cells. In light of the strong link between mutations in FcγRIIB and T1d in both mouse and man, we assessed whether thymic B cells in NOD mice had a MZ or FO phenotype (15). Our data showed that although MZ-like cells are detectable in the thymus of both NOD and control mice, there are significantly fewer in the NOD mouse thymus compared to control animals. It is intriguing that MZ-like cells are present in the thymus of mice, and it will be interesting to see if they function here as surveillance cells against infection or participate in the removal of apoptotic thymocytes.

Nevertheless, our data overwhelmingly ascribes thymic B cells to have a FO phenotype in the NOD mouse, a finding that is in line with several studies in mice and man (33). In NOD mice, CD5, which has been described as a marker for B1a cells in the peritoneal cavity, is also expressed on the thymic FO B cells, although not as extensively as seen in non-NOD strains (29). Thymic FO B cells, in comparison to splenic FO B cells, express much higher levels of MHC class I and II, as well as costimulatory molecule CD40, signalling through which may regulate expression of CD5 (58). Although the majority of thymic B cells in the NOD mice expressed an IgM^+^IgD^+^ BcR, class-switched B cells expressing IgM^-^IgD^-^IgG^+^ BcRs were readily detectable, although notably control mice also had these class-switched cells too. In contrast to this shared phenotype of NOD thymic B cells with non-NOD strains of mice, the NOD thymus harbours class-switched unusual IgM^-^IgD^+^IgA^+^ and IgM^-^IgD^+^IgG^+^ FO B cells where such cells were largely absent in control mice (15). Interestingly, human peripheral B cells with this unusual expression of IgD^+^ in the absence of IgM has recently been described in people living with T1d (59). In people with T1d, these IgD^+^ B cells express polyreactive receptors and can interact with insulin. Although we established that insulin-reactive B cells reside in the thymus of NOD mice, similar numbers of insulin-reactive B cells were present in control, non-autoimmune mice (15). Further, our studies focused on the thymic B cell population in its entirety, not this specific subgroup of B cells. Thus, the antigen specificity of these unique thymic IgD^+^IgG^+^ B cells in NOD mice is yet to be resolved, but potentially harbours an autoreactive BcR repertoire.

## Activation of Thymic B Cells – A Role for Thymic Germinal Centres

B cell activation takes place in specialised germinal centres (GCs) within B cell follicles of secondary lymphoid organs. GCs tend to form in response to infection, although small transient GCs have been seen in non-inflammatory conditions. GC formation requires cross-talk between stromal cells and immune cells, and are integral for the somatic hypermutation, class switching and differentiation of activated B cells into plasma cells and memory B cells reviewed by (60). Aside from conventional GCs, B cell activation can also take place in extrafollicular structures and ectopic GCs. These ectopic GCs occur in non-lymphoid tissue, and result from the remodelling of the tissue stromal cell network in response to inflammation (61). Ectopic GCs are of particular importance in autoimmunity, and several conditions have reported the presence of these structures in the target tissue (62–64), including in the islets of T1d murine models (65), and the presence of ectopic GCs correlates with disease severity. Like conventional GCs, ectopic GCs are compartmentalised into B and T cell areas (64), and several cytokines have been attributed to their localised formation including IL-22 (66) and IL-23 (67). Interferon gamma (IFNγ) seems to be one of the most critical players in ectopic GCs formation (68–70). Despite the remarkable knowledge we now have on conventional and ectopic GCs, little is known about GCs that can form in thymic tissue despite being a characteristic of autoimmune conditions like myasthenia gravis (18, 71), systemic lupus erythematosus (19) and T1d (15). Consistent among the autoimmune conditions where thymic GC occur, the GCs form at the cortical-medullary junction, and in NOD mice, such structures only materialise at the post-insulitic, pre-diabetic phase. Furthermore, the thymus becomes enriched with IL-21 as thymic GCs form, a cytokine that is critical for regulating GC maintenance and promotion of B cell differentiation and proliferation (72). Expression of activation-induced cytidine deaminase (AID) increases, suggesting active somatic hypermutation/class-switching is ongoing. IL-21 has proven a particularly interesting cytokine in promoting T1d both in man (73) and NOD mice (74). At the heart of the link between IL-21 and T1d are the T follicular helper (Tfh) cells (73) and in children at risk of T1d progression, circulating Tfh cell numbers peak around onset of clinical symptoms (75). In man it has been described that Tfh cells within conventional GCs may be identifiable from their recirculating counterpart on the basis of expression of the master transcription factor for Tfh cells- Bcl-6; whereas GC residing Tfh cells express Bcl-6, recirculating Tfh cells may not (76, 77). Nevertheless, circulating Tfh cells are uniquely gifted at entering inflamed tissue, and participating in ectopic GC formation (78) and *in vitro*, can promote B cell class switching retaining characteristics of GC Tfh cells (79, 80). As expected, considering their essential role in GC/ectopic GCs, Tfh cells are enhanced in the thymus of NOD mice at an age when thymic GC formation occurs. These Tfh cells express Bcl-6 and IL-21, as well as other known markers of Tfh cells (15). This suggests that intrathymic Tfh cells may derive from *in situ* CD4_+_ T cells, unless circulating Tfh cells that migrate to the thymus take on the phenotype of GC Tfh cells with respect to Bcl-6 expression.

Certain questions remain about the nature of the thymic GCs in T1d; do they contain follicular dendritic cell structures and what is the source of the inflammation to push thymic GC formation? GCs are populated with specialised follicular dendritic cells (FDC) that act as a depot for antigen presentation within the GC (81). However, we have yet to confirm such cells exist in our thymic GCs (Pinto and Green, unpublished observations). Interestingly, it has been postulated that the type of FDC present in a GC/ectopic GC may be unique to the tissue and type of inflammation (67, 82) and markers of conventional GCs may not be present on thymic FDCs. In terms of inflammation, it could be speculated that viral infections linked to T1d development in man may also infect the thymus (83) or endogenous retroviral infection (84) could induce thymic GC formation. Alternatively thymic GC formation may simply be a reflection of an accelerated ageing of the thymus (85). Indeed, others, have documented accelerated thymic involution in NOD mice (6). It will be important to determine if people who develop T1d also have thymic GCs, and potentially accelerated ageing of this tissue.

## Thymic B Cell Function

The immunohistochemical evidence that thymic B cells form follicles at the cortico-medullary junction, and can form rosette-like structures around T cells in the medulla (86, 87) led to early speculation that thymic B cells were involved in negative selection of autoreactive T cells. Subsequent studies from different groups have substantiated that hypothesis (discussed below), although whether thymic B cells positively or negatively contribute to negative selection seems dependent on the autoimmune nature of the mammal studied. Before we discuss the evidence for the role of thymic B cells in negative selection, let’s first consider their antigen specificity.

We earlier touched on the finding that thymic B cells in NOD mice or control animals expressed receptors for insulin i.e. they were autoreactive. In normal B cell development and maturation, efficiency in removing B cells with autoreactive BcRs is high, with approximately 20% of circulating B cells bearing autoreactive BcRs (88). Interestingly, the removal of B cell with autoreactive BcRs occurs in two stages; initially at the immature B cell stage in the bone marrow and subsequently during the transition of immature B cells to mature B cells following their recent egress from the bone marrow, with the early immature to immature B cell development stage exhibiting the largest removal of self-reactive B cells from the repertoire (88). Thus the earliest stages of B cell development in the bone marrow the repertoire of B cells has a high level of self-reactivity. To determine if the thymic B cell repertoire, similar to bone marrow-derived B cells, had self-reactivity, Rother et al. performed comparative sequencing studies of single cell sorted paediatric thymic B cells versus foetal bone marrow B cells (89). Such studies demonstrated that thymic B cells had a greater specificity for self-peptide autoantigens than similar sequenced foetal bone marrow B cells, these latter cells being more specific for dsDNA. Furthermore, thymic B cells had polyreactivity, recognising multiple autoantigens, including insulin. This prevalence of thymic B cells to harbour an autoreactive BcR has been shown by others (90). It can be envisaged that autoreactive thymic B cells may participate in negative selection by presenting ‘free’ autoreactive antigens- either trafficked to the thymus or captured from dying medullary thymic epithelial cells. Indeed, thymic B cells have been shown to efficiently present antigens their BcR is specific for to developing T cells (91) promoting efficient deletion of autoreactive T cells during T cell development (92–95). However, capture of autoantigens by the thymic B cell autoreactive BcR is not the sole way thymic B cells contribute to negative selection. Yamano et al., using a transgenic autoimmune regulator (AIRE) gene locus encoding a chimeric influenza haemagglutinin protein and human CD2 promoter demonstrated that 50% of thymic B cells express AIRE. In contrast, splenic or bone marrow B cells in the transgenic animal did not (94). Interestingly these AIRE^+^ thymic B cells resided in the medulla, and comparative studies of tissue restricted antigen (TRA) expression between AIRE^+^ thymic B cells and AIRE^+^ medullary thymic epithelial cells found there was no overlap between the two cell types in the TRAs presented. This suggests that thymic B cells may work in concert with medullary thymic epithelial cells to negatively select a greater range of autoreactive T cells. The work of Yamano builds on early reports that thymic B cells deleted superantigen specific T cells and that B cell specific expression of myelin oligodendrocyte glycoprotein enhanced negative selection of MOG-reactive transgenic T cells (16).

This ability of thymic B cells to participate in negative selection is suggested to be linked to their intrathymic class-switching activity (96), autoreactive thymocytes enabling selection and expansion of their cognate autoreactive thymic B cell counterpart. Perera et al., used an AID reporter mouse in a parabiotic model to show that self-antigen can drive class-switching of thymic B cells *in situ*, and the class-switched cells predominantly expressed IgG2b and IgA BcRs (96), and on an autoimmune background, class-switching of autoreactive thymic B cells numbers was enhanced. Further, they showed that impeding class switching of thymic B cells impaired their ability to negatively selective autoreactive T cells.

Others have documented a role for thymic B cells in the development of thymic T regulatory cells (Treg), and animals with expanded thymic B cell compartments have a correlating expanded thymic Treg compartment too (97, 98).

It would appear that there is strong evidence that thymic B cells are important in central tolerance. However, this positive role for thymic B cells in central tolerance is contrasted by the evidence that thymic B cells are key players in mediating tissue damage in myasthenia gravis (18, 63, 71, 99) and more recently systemic lupus erythematosus (19, 100) and T1d (15). Myasthenia gravis is the most documented condition where thymic B cells participate negatively in the autoimmune outcome. In myasthenia gravis, the thymus is the key source for pathogenic acetylcholine receptor antibodies that target this receptor on muscles leading to chronic muscle weakness. The thymus in myasthenia gravis patients have medullary thymic epithelial hyperplasia (18) with autoreactive thymic B cells secreting antibodies to acetylcholine receptors expressed on the medullary thymic epithelial cells triggering their demise *via* Complement-mediated attack (101).

In animal models of systemic lupus erythematosus, thymic B cells have a distinct transcriptome compared to thymic B cells from non-autoimmune prone, with increased prevalence of genes related to B cell survival (19). These increased thymic B cells were shown to promote expansion of the Tfh cells which in turn could enhance the systemic autoantibody response.

Our own studies in NOD mice, suggest that in T1d, thymic B cells may act in a manner similar to that seen in myasthenia gravis. Enhancement of thymic B cell numbers, the formation of thymic germinal centres results in a significant increase in intrathymic antibody levels in contrast to non-autoimmune prone mice. These antibodies are predominantly of the IgG1 and IgA subclass and were unique to the thymic compartment (15). Furthermore, *in situ* binding of these IgG antibodies to thymic medullary epithelial cells correlated with enhanced apoptosis of these antibody-selected cells. We have yet to establish that the antigen recognised by the autoantibodies is insulin, however, we showed that loss of certain medullary thymic epithelial cells resulted in decreased negative selection of autoreactive T cells, and enhanced survival of insulin-reactive thymocytes (15).

**Figure 1:**
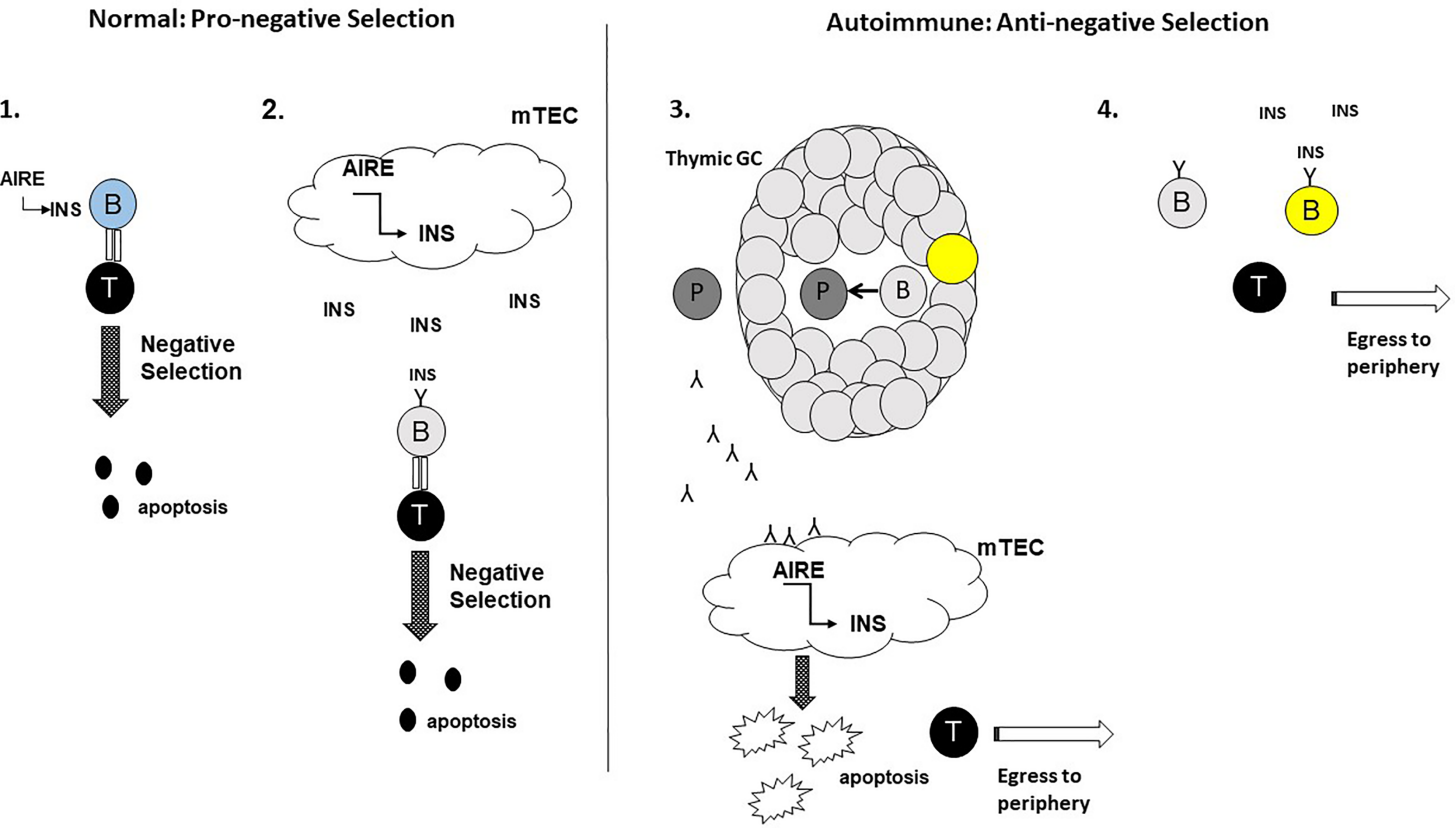
Potential roles for thymic B cells in the thymic negative selection process: a hypothesis. In the normal setting, thymic B cells enhance the negative selection of autoreactive T cells. 1. Aire^+^ Thymic B cells (blue) express and present self-antigens e.g. insulin (INS) to autoreactive T cells with high affinity TcRs for the self-antigens leading to T cell apoptosis. 2. Self-antigens e.g. INS secreted by Aire^+^ mTECs are acquired by thymic B cells expressing self-reactive BcRs (light grey). Internalisation, processing and presentation of the self-antigen to autoreactive T cells leads to T cell apoptosis. In the autoimmune setting, thymic B cells impede the negative selection of autoreactive T cells. 3. The emergence of thymic GCs results in thymic B cells receiving signals to develop into plasma cells (dark grey) secreting autoantibodies for self-antigens expressed by mTECs. Binding of the autoantibodies to mTECs leads to mTEC apoptosis, leading to decreased negative selection of autoreactive T cells and increased egress of the T cells to the peripheral tissues. 4. Somatic hypermutated (yellow) thymic B cells egressing from thymic GCs, may outcompete ‘normal’ thymic B cells for the binding of self-antigen in the thymic milieu impeding negative selection. For this scenario, the sm thymic B cells would either fail to adequately present the self-antigens to autoreactive T cells to support negative selection.

These contrasting roles for thymic B cells in negative selection are intriguing. Whether thymic B cells harbour pro-negative selection and anti-negative selection subpopulations, with the latter population having an advantage over the former in the autoimmune setting remains to be established. Alternatively, it may be that as an autoimmune process is ongoing, pro-negative selection thymic B cells switch to an anti-negative selection functionality. The potential mechanisms that promote these divergent properties of thymic B cells is shown in **Figure 1**. Studies that address these hypotheses will be important in understanding the relationship between thymic B cells and central thymic tolerance.

## Computational Approaches to Develop a Thymic *In Silico* Model

Many challenges face immunologists studying the thymus; early involution of thymic tissue meaning most human studies are based on procured tissue from foetuses, neonates or young children, and acquiring such tissue is not readily accessible due to, among other issues, ethical reasons. Studies of the thymus, particularly the ageing thymus and its many intricacies in cellular cross-talk, requires large cohorts of mice, again rearing questions of ethics. Recent years have seen a drive towards using computational algorithms and *in silico* models to recapitulate the dynamic environments of immune tissues, and assess the role of candidate molecules in a particular pathway. In this last part of the review, we will discuss the potential of developing *in silico* computational models to identify therapeutic avenues for manipulating thymic B cells in autoimmune disorders like T1d.

## *In Silico* Approaches to Modelling Biological Systems

Systems biology is the integration of wet-lab experimentation and computational research in order to understand complex biological systems. Computational biology provides tools for the theoretical exploration of biology, permitting scientists to address critical questions directly (102). Simulation is one facet of computational biology that is finding increasing usage (103–106).

The use of simulation would bypass the necessary ethical, financial and practical issues surrounding acquisition of thymic tissue. In addition, the very nature of the system lends itself very precisely to Agent Based Modelling and Simulation (ABMS); consisting as it does of very many heterogeneous individuals of different types e.g. B cells, T cells, thymic medullary epithelial cells. These cell populations can interact with each other in specific ways under specific conditions. The cells will also possess some concept of location (the medulla or the cortico-medullary junction) and state (e.g. some stage of the cellular life cycle). Use of agent-based modelling will permit us to investigate how manipulating cell behaviours will give rise to altered system-wide (‘emergent’) behaviour. The results will be easily interpretable and so simple to put into words accessible to a wider audience.

## Mathematical Versus Computational Models

A simulation is typically either mathematical or computational. Mathematical models are generally based on series of differential equations (107) and cells are modelled as populations rather than as individuals (108). Such models are often seen as opaque to non-mathematicians (109) and the model is typically difficult to extend should further cell types need to be considered. A further perceived disadvantage of mathematical models is that the resulting differential equations tend be complicated to solve exactly and often require solving *via* numerical methods which entails potentially unacceptable approximations to the model.

On the other hand, computational simulation methodologies such as Agent Based Modelling (ABM) are typically conceptually much simpler (110, 111). Cellular populations are modelled as sets of individuals (112) that share similar behaviours e.g. a population of Single Positive T cells. In this way more descriptive explanations of experimental observations may be proposed from simulation outcomes. Additionally agent-based models are better suited than other modelling techniques to capturing the system-wide, or emergent, behaviour of the system (113). Here, emergent behaviour is understood to be the system behaviour arising from the combined behaviours of the component entities e.g. cells.

An ABM will represent some abstraction of the system created jointly by a scientist, expert in the system of interest, and a software developer. The model should aim to include all factors e.g. cells and signalling molecules, generally held to be essential to system function. It is also important to include any potential roles for the biological environment in the model.

## Creating an Agent-Based Model

A number of short tutorials on the creation of agent based models can be found in the literature e.g. Bandini et al., (114). Generally, the principal steps in developing an agent-based simulation are:

- Identify the agents that are important to what you want to model:

For example in a model of negative selection events in the thymic medulla, we might wish to model the behaviours of Single Positive T cells, B cells and medullary thymic epithelial cells (mTECs).

ii. Identify the environment in which your agents exist:

We might for instance break the thymus into three distinct environments: the cortex, the cortico-medullary junction (CMJ) and the medulla; placing relevant cells in each.

iii. Identify mechanisms whereby the agents interact with each other and with their environment:

We might consider that cells behave differently in different environments. For example, SP T cells will not tend to remain in the cortex, but will rather migrate across the CMJ to the medulla where they will interact with mTECs, *via* recognition of the insulin fragments presented by the mTECs, to facilitate negative selection. Aggressive B cells will also be able to interact directly with mTECs, also *via* recognition of insulin presented by the mTECs.

iv. Consider how best to implement the model as computer code and any assumptions and simplifications necessary in achieving this:

It will be very difficult to exactly replicate precise biological behaviour as computer code. For example, cells are unlikely to be of regular shapes e.g. circular, so the geometry of the system will not be exactly reproducible *in silico* and appropriate approximations will be necessary. In the case of thymic B cells it will be necessary to decide how we will differentiate between those B cells which serve to enhance the negative selective of autoreactive T cells and those that prevent the negative selection of autoaggressive T cells.

## Challenges and Limitations of the Approach

Despite the benefits of employing simulation as an investigative tool, it is wise to be mindful of the potential pitfalls in the use of the technique.

As an experiment, a simulation must be reproducible. It is therefore of paramount importance that simulation design be comprehensively documented. The documentation must incorporate all assumptions and design decisions to make them available to the scientific community to assess. The CoSMoS simulation design protocol (115) provides guidance in documentation and development of simulations, in which the user can have confidence.

The first challenge in the design of agent-based models is to correctly capture the relevant entities (cells, signalling molecules etc.) of the system and their behaviours in the model. If a key component of the system (or its behaviour) is omitted from the model then the model will be unfit for the purpose for which it was designed. As an example, two different types of behaviour in the B cell population have been noted. Some cells appear to be essentially quiescent, though may play an active role in sustaining negative selection, whereas others adopt a more aggressive role, apoptosing mTECs and thus triggering the breakdown of negative selection and hence central tolerance. Effective communication between the software developer and the expert biologist will help to mitigate this problem.

Also, simulation outcomes will be determined by the choice of values for the required simulation parameters. Model parameters may represent quantities such as the duration of a particular cell cycle stage or the concentration of cytokine that brings about progression to the next differentiation state. Such values will, in all likelihood, not be available from experiment and must be estimated. Chosen parameter values will ultimately impact on overall system behaviour and simulation output. Calibration is the process of adjusting parameter values so as to align *in silico* simulation results with observed *in vivo* behaviours. The issues surrounding simulation parameterization are discussed briefly below, but are addressed more fully in the relevant literature:

Optimization of parameter values is the subject of numerous widely used techniques such as the Latin Hypercube (116). This technique is based on the random sampling of the entire parameter space and is time and resource intensive to perform for a typical biological system.

The stability of simulation performance with parameter perturbation can be assessed using two different types of analysis. Robustness analysis is used to gauge the impact of simulation parameters on the simulation’s ability to execute stably i.e. which parameter values might cause the simulation to crash (117). Sensitivity Analysis can be used to investigate the effects of individual parameters or combinations of parameters on simulation outputs *via* the systematic variation of parameter values and observation of the effect on simulation performance (117). Sensitivity analyses are further discussed in (118, 119).

Recent research has aimed to refine techniques that facilitate calibration of simulations involving large numbers of parameters in a less resource intensive manner than the Latin Hypercube method described above. These novel techniques include the use of Machine Learning and multi-objective calibration (120, 121).

Although the scope and particularly the calibration of computational simulations is potentially limited by the scale of the computational resources available, there is a trend towards easier access to powerful high performance computer clusters which makes these concerns less relevant.

Despite the challenges entailed in employing an ABMS approach to biological simulation, ABMS remains a highly descriptive and powerful methodology for elucidation of cellular and molecular mechanisms of disease and many of the challenges posed by the use of the technique, particularly those relating to parameterization, are becoming more easily addressed using recent developments.

## Final Comments

Commonalities in autoimmune conditions offer insights into novel therapies that may target multiple autoimmune conditions. Evidence that abnormality in the thymic B cell compartment is a shared characteristic between several autoimmune conditions highlights the need for further studies into this enigmatic cell. Nevertheless, increasing ethical considerations and availability of thymic tissue for analysis makes studies of thymic B cells somewhat challenging, particularly in man. Computational models of the dynamic thymic environment may offer a new approach to address the role of thymic B cells in health and disease, leading to candidate molecules or signal pathways as novel therapeutic targets in autoimmune conditions, including T1d.

## Author Contributions

DC and EAG wrote the thymic B cell section. RG wrote the computational modelling section. All authors contributed to the article and approved the submitted version.

## Funding

This work was supported by a Diabetes UK Project Grant 18/0005804 awarded to EAG, and a Daphne Jackson Trust Fellowship awarded to RG. The University of York and the Medical Research Council funded the Fellowship.

## Conflict of Interest

The authors declare that the research was conducted in the absence of any commercial or financial relationships that could be construed as a potential conflict of interest.

## Publisher’s Note

All claims expressed in this article are solely those of the authors and do not necessarily represent those of their affiliated organizations, or those of the publisher, the editors and the reviewers. Any product that may be evaluated in this article, or claim that may be made by its manufacturer, is not guaranteed or endorsed by the publisher.
